# Development and Characterization of Gel-Based Buccoadhesive Bilayer Formulation of Nifedipine

**DOI:** 10.3390/gels9090688

**Published:** 2023-08-26

**Authors:** M. Alagusundaram, Nem Kumar Jain, M. Yasmin Begum, S. Angala Parameswari, Vinod Kumar Nelson, Mohammad F. Bayan, Balakumar Chandrasekaran

**Affiliations:** 1 Department of Pharmaceutics, School of Pharmacy, ITM University, Gwalior 474001, Madhya Pradesh, India; 2 Department of Pharmacology, School of Pharmacy, ITM University, Gwalior 474001, Madhya Pradesh, India; nemjain.pharma@itmuniversity.ac.in; 3 Department of Pharmaceutics, College of Pharmacy, King Khalid University, Abha 61421, Saudi Arabia; 4 Department of Pharmaceutical Analysis, Jagan’s Institute of Pharmaceutical Sciences, Nellore 524346, Andhra Pradesh, India; eswarialagusundaram@gmail.com; 5 Department of Pharmaceutical Chemistry, Raghavendra Institute of Pharmaceutical Education and Research, Anantapuramu 515721, Andhra Pradesh, India; vinod.kumar457@gmail.com; 6 Faculty of Pharmacy, Philadelphia University, P.O. Box 1, Amman 19392, Jordan; mbayan@philadelphia.edu.jo (M.F.B.); balakumar@philadelphia.edu.jo (B.C.)

**Keywords:** nifedipine, buccoadhesive, triggered delivery, ex vivo permeation, polymeric gel

## Abstract

A promising controlled drug delivery system has been developed based on polymeric buccoadhesive bilayered formulation that uses a drug-free backing layer and a polymeric hydrophilic gel buccoadhesive core layer containing nifedipine. The DSC thermogravimetric analysis confirms the drug’s entrapment in the gel layer and reveals no evidence of a potential interaction. Various ratios of bioadhesive polymers, including HPMC K100, PVP K30, SCMC, and CP 934, were combined with EC as an impermeable backing layer to ensure unidirectional drug release towards the buccal mucosa. The polymeric compositions of hydrophilic gel-natured HPMC, SCMC, and CP formed a matrix layer by surrounding the core nifedipine during compression. Preformulation studies were performed for all of the ingredients in order to evaluate their physical and flow characteristics. Ex vivo buccoadhesive strength, surface pH, swelling index, in vitro and in vivo drug release, and ex vivo permeation investigations were performed to evaluate the produced gel-based system. Rapid temperature variations had no appreciable impact on the substance’s physical properties, pharmacological content, or buccoadhesive strength during stability testing using actual human saliva. It was clear from a histological examination of the ex vivo mucosa that the developed system did not cause any irritation or inflammation at the site of administration. The formulation NT5 was the best one, with a correlation coefficient of 0.9966. The in vitro and in vivo drug release profiles were well correlated, and they mimic the in vitro drug release pattern via the biological membrane. Thus, the developed gel-based formulation was found to be novel, stable, and useful for the targeted delivery of nifedipine.

## 1. Introduction

The main goal of any drug delivery system is to deliver the medication precisely and efficiently to the body’s site of action. This system should be able to trigger and control the drug release at the intended spot, increasing drug availability, increasing therapeutic efficacy, minimizing potential side effects, and reducing dose frequency [1]. The buccal portion of the mouth cavity is a desirable target for medication delivery [2], and the administration of the desired medication via the buccal mucosal membrane lining of the mouth cavity is known as buccal delivery.

The mucosal lining of buccal tissues provides a significantly more hospitable environment for drug absorption than oral drug delivery, with the latter presenting a hostile environment for medications, especially proteins and polypeptides, due to acid hydrolysis and the hepatic first-pass effect [3,4]. By overcoming the limitations of traditional administration routes, the buccal mucosa is a valuable channel for treating either local or systemic medicines. The buccal (inside the cheeks), sublingual (under the tongue), and gingival (on the gums) areas of the oral cavity are the places where medications are administered [5,6]. The best areas for medication delivery are the buccal and the sublingual sectors, which can be used to treat either systemic or local disorders. The buccal mucosa is a well-vascularized, easily accessible tissue for both administering and removing a delivery device [7,8]. This involves the ability to construct poly or single directional release systems for local or systemic activities, and to incorporate permeability enhancers, enzyme inhibitors, or pH modifiers in the formulation [9,10]. The oral mucosa is well supplied with blood. After being absorbed from the oral cavity by the oral mucosa, drugs reach the systemic circulation via the deep lingual or the facial vein, the internal jugular vein, and the brachiocephalic vein [11]. Following buccal delivery, the drug enters the bloodstream directly, thereby skipping the first-pass effect.

**Figure 1:**
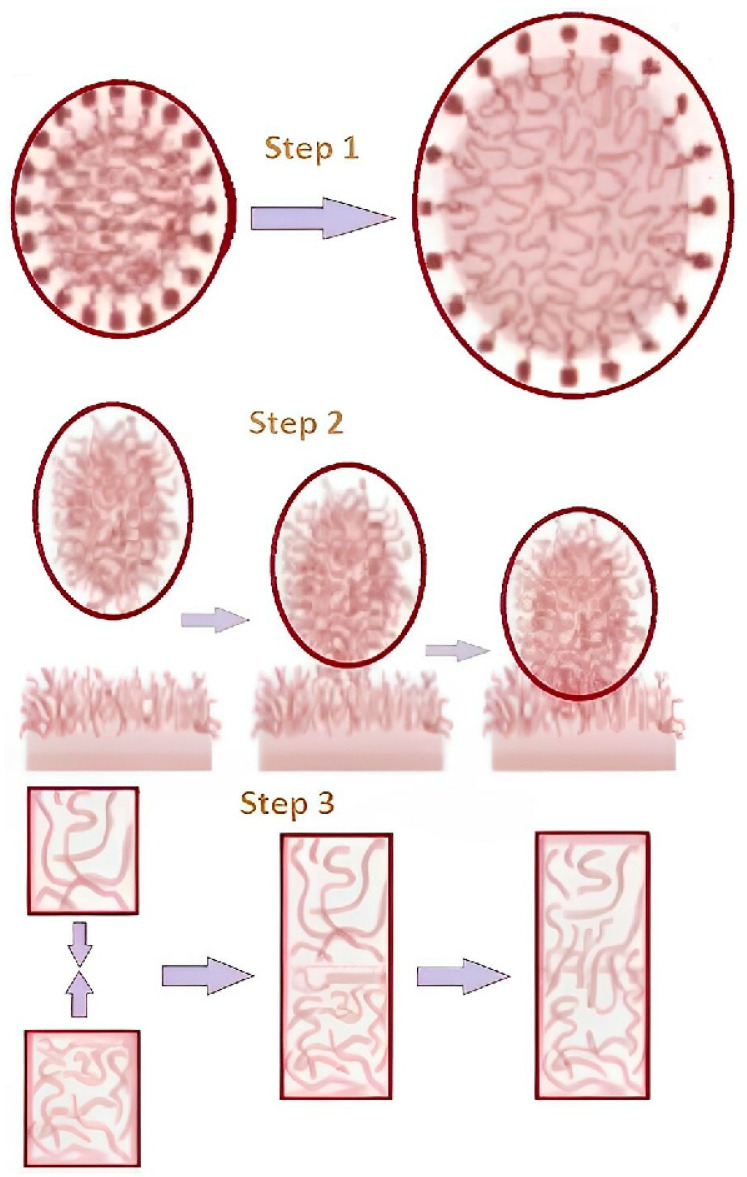
Mechanism of bioadhesion includes wetting and swelling, interpenetration and formation of chemical bonds.

The three-dimensional polymeric networks of hydrophilic polymeric gel can absorb large to small amounts of active pharmaceutical ingredients, and, as a result, they are widely used to deliver medicines that are more biocompatible and have less discharge [12]. The main reason that HPMC (Hydroxy Propyl Methyl Cellulose), SCMC (Sodium Carboxy Methyl Cellulose), and CP (Carbopol) are so good at absorbing water is because their molecular chains have hydrophilic groups, such as amino, amide, hydroxyl, and carboxyl groups [13]. Polymeric hydrophilic gel systems have been shown to have different physicochemical parameters, such as porosity, structural softness, swelling capacity, and elasticity, because they have these hydrophilic groups. Bioadhesion occurs when two surfaces, one of which must be a mucous tissue or membrane, come into contact with a polymeric hydrophilic gel-based matrix layer, and this is the ability of a material to adhere to the surface of the buccal mucosal layer [14]. When glandular columnar epithelial cells release mucosal layers, they primarily consist of mucin and water with trace amounts of lipids, other proteins, and muco-polysaccharides. More specifically, when thinking about epithelial tissue or the mucus coat on the surface of a tissue, the connection to a mucus coat is formed by mucoadhesive gel in a hydrophilic environment. The mechanism of the bioadhesion process occurs in three steps: the wetting and swelling of the polymer to form a hydrophilic gel by absorbing the mucin (Step 1), the interpenetration of the gel chains and the mucosal membrane (Step 2), and there chemical bond formation between the entangled chains (Step 3) [15]. The schematic illustrations of the above steps are represented in Figure 1.

Nifedipine is a calcium channel blocker of the dihydropyridine type, and it is mainly used to treat hypertension and angina pectoris [16]. Due to its short elimination time of 2–4 h, nifedipine is a good choice for controlled release delivery. The primary objective of this study was to formulate and characterize polymeric buccoadhesive gel-based bilayer formulations that would release nifedipine in a unidirectional pattern at the site of administration for an extended period of time without being washed away by saliva.

## 2. Results and Discussions

The purpose of this research was to both produce and evaluate polymeric buccoadhesive gel-based bilayer formulations that deliver the drug in a unidirectional pattern at the site of administration over time. Using HPMC-K100M, SCMC, PVP-K30, and CP934, the bilayer gel-based systems were created through direct compression [17,18]. Because of its minimal water permeability and its flexibility in the buccal environment, EC was chosen as the backing layer [19]. The HPMC, SCMC, and CP form a gel coating over the nifedipine nucleus. When these polymers are hydrated with water, they form viscous gels with a long duration of retention on mucosal surfaces, resulting in the formation of adhesive contacts [20]. In conjunction with an increase in the concentration of the HPMC, a change in the morphology of nifedipine from cubical to plate-like was observed, and this led to an increase in the dissolution rate during the in vitro release experiments.

### 2.1. Drug Polymer Interaction Studies through DSC

**Figure 2:**
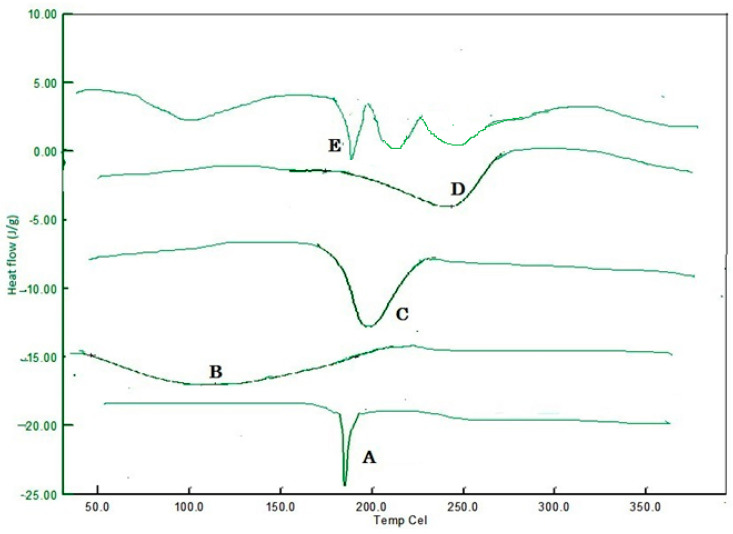
DSC thermogram: (A) Nifedipine (173.0 °C); (B) HPMC K 100 (112.2 °C); (C) SCMC (189.0 °C); (D) CP 934 (241.0 °C); (E) Nifedipine-polymer mixture.

DSC is used for investigation into any physicochemical interactions that may exist between the drug and the matrix polymer. Thermal analysis of nifedipine alone and also in the presence of various polymers is performed in an incremental series of weight fractions. Thermogram of the first run indicated that the melting point of pure nifedipine is approximately 173 °C as an endothermic peak. Due to the dehydration process of the HPMC, a large, broad endothermic effect was observed over a temperature range of 100 to 120 °C, with a shift to 112.2 °C as a larger endothermic peak [21]. The difference in the chemical composition of cellulose in the SCMC causes the observed difference in thermal decomposition behavior and thermal stability. The thermogram of the SCMC shows the fraction of weight loss as 80.4%, indicating that the neat cellulose and the SCMC contains the fraction of non-volatile components. The thermal decomposition process of cellulose occurs approximately 189 °C as an endothermic peak. The thermogram of carbopol shows a broad endothermic peak at 241 °C, which indicates the evaporation of adsorbed water on CP 934, and, after, the melting point decomposition begins. The DSC diagram of the physical mixture of nifedipine-polymer mixture shows a similar sharp well-defined endothermic peak of nifedipine, which indicates that no interaction persists between the drug and polymer. The DSC thermogram of nifedipine, HPMC K 100, SCMC, CP 934, and nifedipine-polymer mixture is shown in curves A, B, C, D, and E in Figure 2, respectively, and is individually shown in Figures S1–S5, respectively.

### 2.2. Physicochemical Evaluation of Buccoadhesive Bilayered Formulations

Following the procedures that are outlined in the pharmacopoeia, the prepared gel-based formulation was characterized for thickness, weight variation, hardness, and friability in order to determine the stability of the product during transportation, packaging, and storage. All of the formulations revealed an acceptable thickness, weight uniformity, rigidity, friability, and drug content as per the accredited pharmacopoeia. In order to estimate the amount of the drug to be used, the prepared formulations were evaluated for drug content in triplicate, and the drug content was found to be in a range between 19.78 ± 0.22 to 20.32 ± 0.21 mg. Using polymers, the hardness of the formulations was maintained within a range of 4.0 to 4.3 kg/cm^2^. Concentrations of the HPMC and film-forming PVP resulted in a marginal increase in hardness. Changes in hardness had no significant effect on the release profile of the nifedipine. The observed results indicated reproducibility with minimum intra-batch variability. The formulation NT5 comprised of HPMC K100, and PVP K30 was discovered to have a hardness of 4.2 kg/cm^2^. Due to its strong and viscous gel layer, HPMC is frequently used for the preparation of hydrophilic matrix gels [22]. This layer shields the matrix from rapid disintegration, and it regulates the rate of drug release without altering the release profile. Compared to all of the other formulations, NT5 exhibited superior performance across all of the physicochemical parameters. The post-compressional evaluation results are presented in Table 1 below.

#### Surface pH

The surface pH of the produced gel-based formulations was determined in light of the fact that an acidic or alkaline pH can irritate the buccal mucosa and can affect the rate of hydration of polymers [23]. The observed surface pH range for the samples was 6.68 to 6.80. These results demonstrate that there is no significant difference in the surface pH of any of the formulations, and that the pH range falls within the range of salivary pH, i.e., 6.5 to 6.8, which does not cause irritation, and which thereby assists patient compliance. Interaction between the hydroxyl groups of HPMC and the carbonyl groups of PVP in formulation NT5 occurred. This system either disperses or interacts with hydrophobic nifedipine, whereas PVP is thought to inhibit nifedipine-HPMC by interacting with the co-crystal surfaces, which results in a pH close to neutral or a salivary pH of 6.8.

### 2.3. Swelling Index

**Figure 3:**
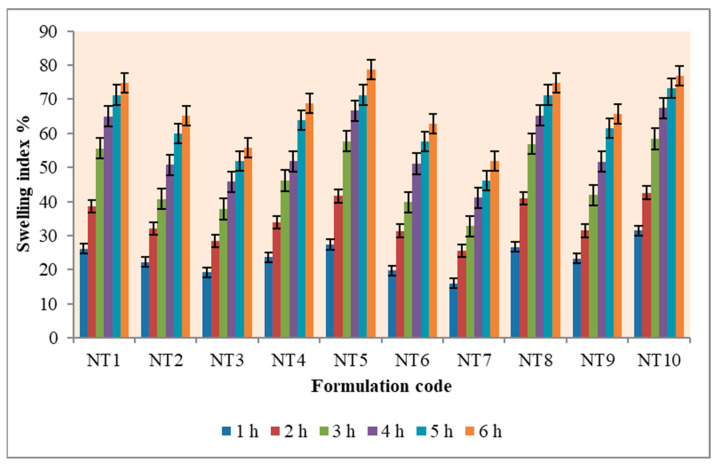
Swelling index % of NT1-NT10.

Based on reports, the swelling behavior of the gel is crucial to its bioadhesive properties and its drug release profile [1]. The interaction between the hydroxyl groups of HPMC and the carbonyl groups of PVP improves the mucoadhesive properties of the formulations in comparison to those of the unaltered HPMC. The adhesion increases with the degree of hydration until the point of disentanglement at the surface of the gel tissue, and this results in a sudden decrease in adhesive strength due to overhydration. The adhesion is initially strong, but it progressively weakens after swelling, and can last for up to six hours. The swelling index increased as the formulation weight gain increased proportionally with the rate of HPMC hydration. The formulation NT5 with the highest concentration of HPMC and carbopol had the highest index of swelling after 6 h. The results are represented in Figure 3 and Table S1.

### 2.4. Ex Vivo Buccoadhesive Strength

**Figure 4:**
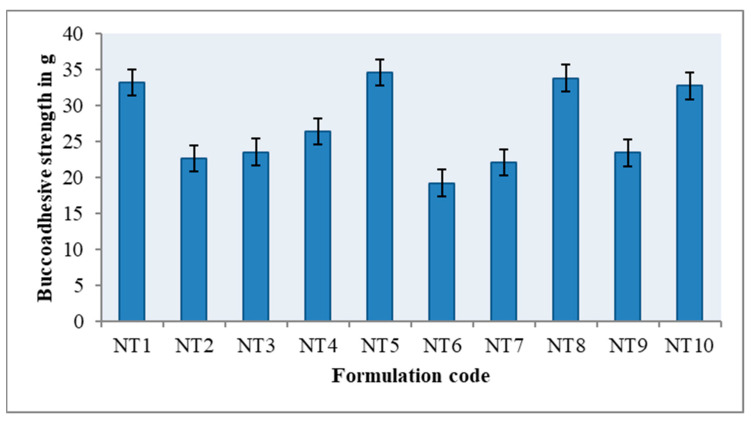
Ex vivo buccoadhesive strength of NT1-NT10.

With a modified physical balance method and fresh buccal mucosa from a nearby slaughterhouse, the ex vivo buccoadhesive strength was measured [24]. The carboxyl and hydroxyl functional groups of HPMC make it easier for hydrogen bonds to form between the gel and the mucous membrane. The presence of more hydrophilic functional groups facilitates the formation of more hydrogen bonds. A gel’s molecular structure determines its level of hydration. Hydration is necessary for the gel to enlarge on the mucus layer, maximizing exposure for the gel and mucin interpenetration, and providing entanglement between them [25]. The carbonyl groups of PVP reduce the excess hydration of HPMC, and they increase its mucoadhesive properties. In addition to its mucoadhesive properties, HPMC is also widely used for its controlled release mechanism. The observed buccoadhesive strength may be satisfactory in all formulations, and this ensures that all of the samples are successfully maintained in the buccal cavity. The results are illustrated in Figure 4 and Table S2. Due to the miscibility of HPMC and PVP, the formulation of NT5 possesses substantial buccoadhesive strength.

### 2.5. In Vitro Drug Release Study

In all of the formulations, the release of nifedipine differed significantly, and this may be attributable to the variable proportions of polymeric substances. The formulations produce a reasonable nifedipine discharge at the end of 12 h. Formulations NT1, NT2, NT3, NT4, NT5, and NT6 containing combinations of HPMC, PVP, SCMC, and carbopol gave a reasonable Nifedipine release of up to 12 h. The constituents of NT1, NT2, NT3, NT4, NT5, and NT6 demonstrated respective rates of release of 98.3%, 97.1%, 97.4%, 98.6%, and 97.6%. The regression coefficient (R), Higuchi’s plot, and the in vitro drug release all showed that the drug release followed zero-order kinetics. Peppa’s plot showed that the diffusion exponents (n) were 0.93067, 0.85066, 0.89323, 0.85197, 0.91961, and 0.89929, respectively. This shows that the drug release mechanism was non-Fickian release for NT2 and NT4, and Super Case II transport type for NT1, NT3, NT5, and NT6. The release of Nifedipine from NT7-NT10 with HPMC, SCMC alone, or a combination of HPMC, SCMC, PVP, and Carbopol, was good for up to 12 h. The release rates of NT7-NT10 were 97.4%, 98.4%, 98.2%, and 98.1%, respectively. The release rate of nifedipine was dependent on the swelling index and buccoadhesive strength, which can vary depending on the characteristics and composition of matrix-forming gels. In general, the rate of drug release increased as the proportion of hydrophilic polymer increased. Due to increases in swelling index and buccoadhesive strength, it was possible to attribute the maximal cumulative percentage release of nifedipine from formulation NT5 to proportions of HPMC, PVP, and CP.

**Figure 5:**
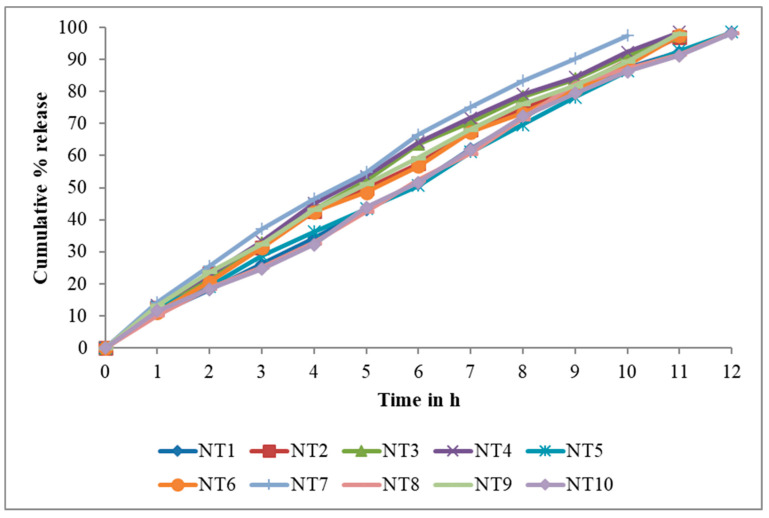
In vitro drug release data of NT1 to NT10.

**Figure 6:**
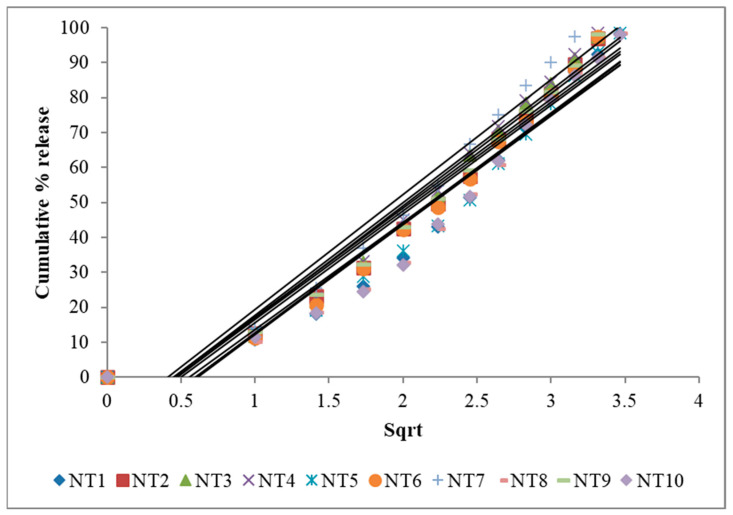
Higuchi’s plot of NT1 to NT10.

**Figure 7:**
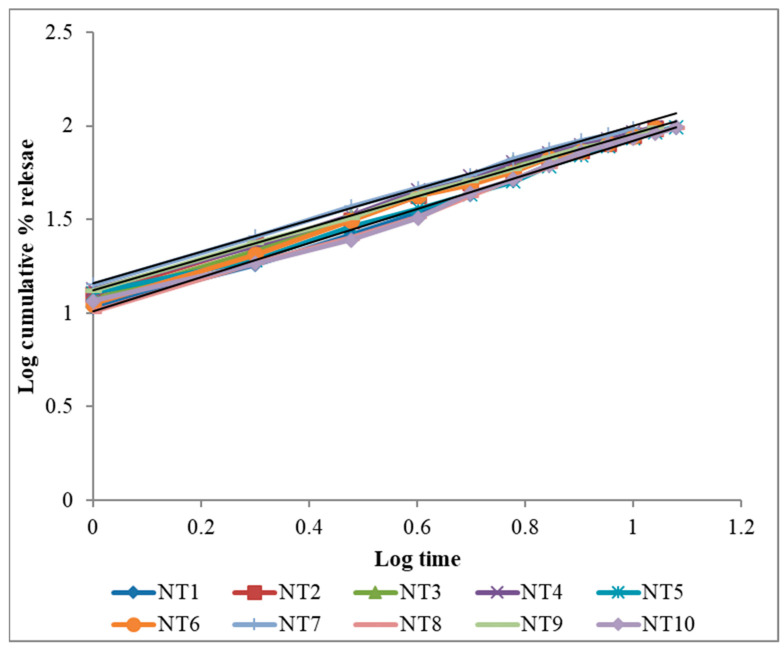
Peppa’s plot of NT1 to NT10.

The in vitro drug release (Figure 5) demonstrated that the drug release followed zero-order kinetics, which was supported by the regression coefficient (R). Higuchi’s plot (Figure 6) as cumulative % drug released versus square root of time, and the obtained values were presented in the Table S3. Peppa’s plot (Figure 7 and Table S4) was drawn, revealing slope values of 0.8421, 0.94699, 0.83522, and 0.85655, respectively, for NT7 to NT10, confirming that the diffusion mechanism involved in the drug release was non-Fickian release for formulations NT7, NT9, and NT10, and Super Case II transport type for NT8. With the addition of HPMC, SCMC, PVP, and Carbopol bilayer buccal formulations, maximal drug release was observed at the end of 12 h.

To determine the mechanism of drug release from hydrophilic matrices, in vitro dissolution data of each formulation were analysed in conjunction with various kinetic drug release equations [26,27]. To be precise, these are zero order: Q = K0t; Higuchi’s square rate at time: Q = KHt1/2 and Peppa’s: F = Km^tn^, where Q is the amount of drug release at time t; F is the fraction of drug release at time t; K0 is the zero-order kinetic drug release constant; KH is Higuchi’s square root of time kinetic drug release constant; Km is a constant incorporating the geometric and structural characteristics of the polymeric gel; and n is the diffusion exponent indicative of the release mechanism. The values of the correlation coefficient (r^2^) indicate that the kinetics of drug release was zero. The mechanism of drug release was determined using Peppa’s model, which indicates super case II transport based on diffusion exponent values (n).

### 2.6. Ex Vivo Permeation Studies

**Figure 8:**
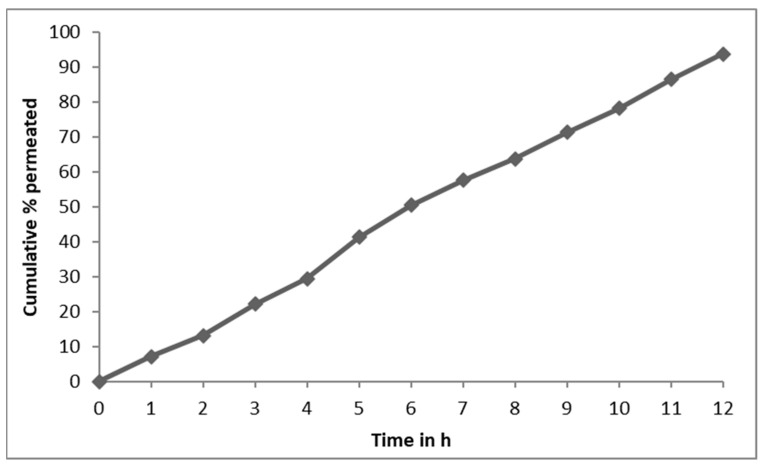
Ex vivo permeation studies of NT5 through sheep buccal mucosa.

Oral mucosa constitutes a barrier to drug permeation, and its permeability characteristics are intermediate between those of the skin epidermis and the gastrointestinal tract. Ex vivo permeation studies can determine the efficacy of the buccal barrier and whether buccal absorption could be used to administer NT5. The formulation, NT5, was subjected to permeation tests and the results presented in Figure 8 and Table S5. The results of drug permeation of nifedipine through the sheep buccal mucosa showed that the drug was released from the formulation and permeated through the buccal membrane. This suggests that the drug could possibly permeate through the human buccal membrane. Ex vivo permeation studies can determine the effectiveness of the buccal barrier and whether buccal absorption can serve as a route of administration for nifedipine. Overall, the permeability studies have helped figure out how well the formulation NT5 absorbs nifedipine, how it moves across the human buccal mucosa, and how buccoadhesive bilayered formulations make permeability higher.

### 2.7. Ex Vivo Muco Irritation by Histological Examination

**Figure 9:**
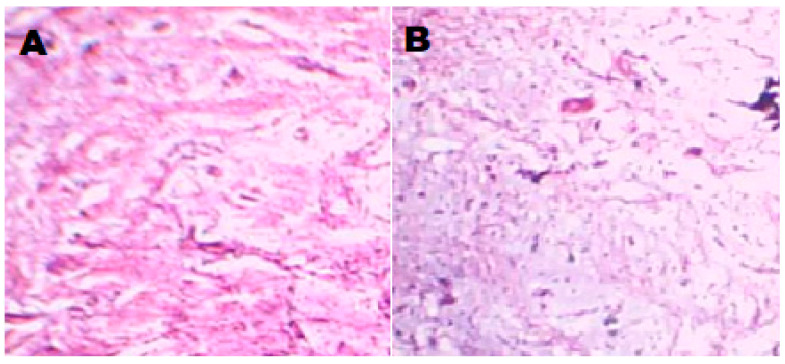
Controlled untreated (**A**) and nifedipine buccoadhesive bilayer gel treated buccal mucosa (**B**).

During the administration of buccoadhesive samples, pathological alterations in cell morphology and tissue organisation were evaluated via histological analysis. Observations under a microscope indicate that NT5 has not significantly damaged the structure of the buccal mucosa. Following permeation examinations, neither cell necrosis nor complete removal of the epithelium from the buccal mucosa was observed. There were no indications of irritation, such as vascular congestion and subepithelial oedema. Furthermore, no severe indicators, such as epithelial necrosis, epithelial cell shedding, or bleeding, were detected on any surface of the buccal mucosa. Neither the basal membrane nor the superficial portions of the submucosa were altered in comparison to untreated mucosa. Figure 9 depicts the resulting images for untreated and treated buccal mucosa as well as Figures S6 and S7.

### 2.8. In Vivo Drug Release Study

**Figure 10:**
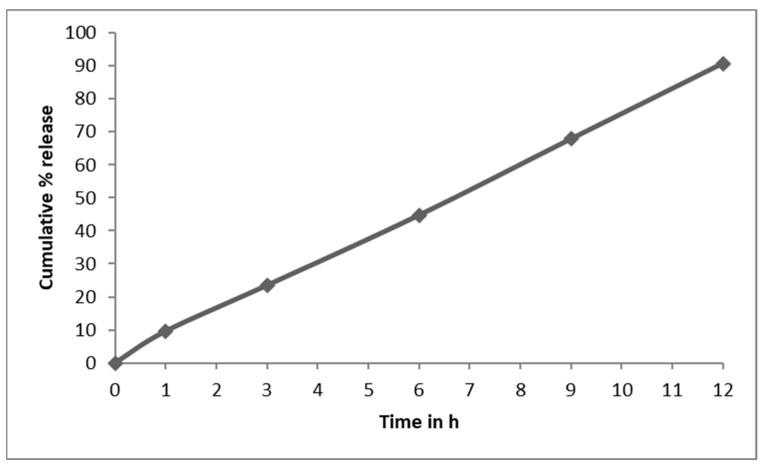
In vivo drug release of NT5.

In rabbits, in vivo buccal diffusion investigations for NT5 revealed a zero-order release pattern. When nifedipine buccoadhesive formulations were given to rabbits in real life, there was no evidence of inflammation, irritation, or other sensitization at the site where the medicine was given. The representations are shown in Figure 10 and Table S6.

### 2.9. In Vitro—In Vivo Correlation

**Figure 11:**
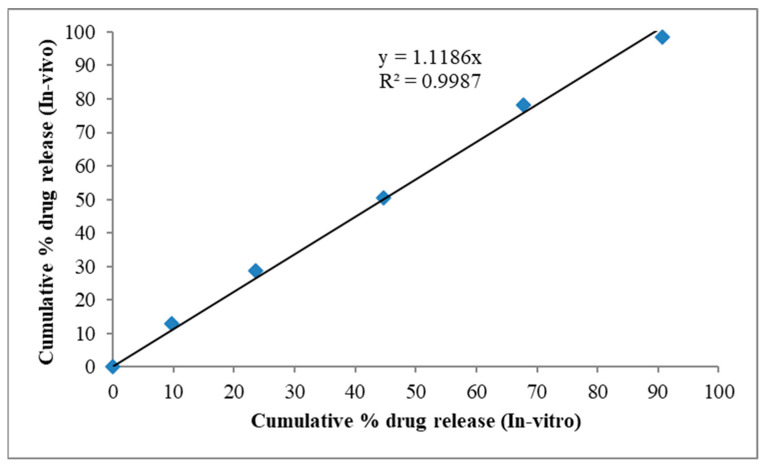
IVIVC plot Stability study in human saliva.

Correlations between in vitro and in vivo drug properties were used to show that the therapeutic effectiveness of buccoadhesive nifedipine formulations is based on both in vitro and in vivo drug properties. Figure 11 shows a graph with cumulative% in vitro release on the x-axis and cumulative% in vivo drug release on the y-axis for the same period of time and the values represented in Table S7. The release rate followed zero order, and the correlation coefficient value was 0.9966.

### 2.10. Stability Study

Stability studies should evaluate product properties that are susceptible to change during storage and that are anticipated to influence quality, safety, and efficacy. The stability studies of NT5 were carried out at accelerated conditions of 40 ± 2 °C 75 ± 5% RH over a three-month period, and periodically checked for appearance, buccoadhesive strength, and in vitro drug release. The result was analysed by one-way ANOVA followed by Tukey’s test, which indicates that the sample was stable and that the *p* value is non-significant. The results are shown in Table 2. It was demonstrated that neither the physical appearance nor the buccoadhesive strength nor the in vitro drug release changed.

## 3. Conclusions

The HPMC K 100, SCMC, PVP K 30, and carbopol 934 polymers, along with an impermeable backing layer of ethyl cellulose, were directly compressed to produce nifedipine buccoadhesive bilayer formulations with a polymeric hydrophilic gel-based matrix. The DSC thermogravimetric analysis confirms the drug’s incorporation into a polymeric structure with only a physical process and reveals no evidence of a potential interaction. The physicochemical characteristics of the produced samples, such as surface pH, swelling percentage, thickness, weight fluctuation, hardness, friability, and drug content, all adhere to pharmacopoeial standards for all samples. Reproducible findings were obtained in rabbits for ex vivo buccoadhesive strength, in vitro drug release, ex vivo permeability, and in vivo drug release. Histological analysis of ex vivo mucosa reveals that the sample did not produce any irritation or inflammation at the delivery site. The best formulation overall was NT5, which comprises 25 mg of HPMC, 10 mg of CP, and 12.5 mg of PVP. With a correlation value of 0.996, the in vitro and in vivo drug release profiles were well correlated, demonstrating the capacity of the system to mimic the in vitro release pattern via the biological membrane. The reduced dose frequency suggests that the proposed gel-based formulation could be used as a viable controlled drug delivery strategy for nifedipine.

## 4. Materials and Methods

Nifedipine was bought from Drugs India in Hyderabad, India, along with HPMC K 100, PVP K 30, SCMC, CP 934, and EC. Newly collected buccal mucosa of sheep for testing the strength of the buccoadhesive and ex vivo permeation studies was obtained from a local slaughterhouse in Gwalior, India. All other supplies obtained and utilized were of an analytical standard. By using direct compression, the buccoadhesive bilayer gel-based formulations were produced.

### 4.1. Drug Polymer Interaction Studies through Differential Scanning Calorimetry (DSC)

DSC is utilized to investigate any physicochemical interaction between the Nifedipine and the polymer matrix [28]. The DSC thermograms of pure drug, polymer, and the composition of drug polymers were recorded in the DSC analyzer model Universal V4.5A at a heating rate of 20 °C per minute from 0 to 350 °C in a nitrogen environment. The nonexistence of the drug melting peak in the DSC thermogram is typically indicative of the substance’s porous or crystalline state in the polymer. Any sharp or significant alteration in the thermal behavior of both the drug and the polymer also manifested as movements of exothermic and endothermic peaks, and these alterations are typically attributed to drug and polymer interactions.

### 4.2. Preparation of Buccoadhesive Bilayer Formulations of Nifedipine

Nifedipine containing bilayer buccoadhesive formulations were created using the direct compression technique [29]. Different sets were made to find the best formulation by adjusting the HPMC, SCMC, and PVP K 30 ratio. The medication was combined with the mucoadhesive polymers HPMC, SCMC, PVP K-30, CP, mannitol, and lactose in a glass mortar, and mortared for fifteen minutes. The powder was completely mixed and screened through a 60 m sieve before direct compression. Magnesium stearate was used to lubricate the mixture for three to five minutes. The combination (100 mg) was then squeezed in a nine-station rotary punching machine (Ahmadabad, India) using an 8 mm diameter die. The impermeable EC backing layer was placed on the compact above, and the upper punch was elevated. The two layers were then crushed into a buccoadhesive bilayer gel. The components of the Nifedipine bilayer buccal formulations are listed in Table 3.

### 4.3. Physicochemical Evaluation of Buccoadhesive Bilayered Formulations

Following the technique outlined for conventional oral formulations in the certified pharmacopeia, all of the manufactured formulations were assessed for post-compressional evaluations, such as thickness, weight variation, hardness, friability, and drug content [30].

#### Surface pH

To check for any potential side effects in the buccal environment, the surface pH of the buccal formulations was measured. It was decided to maintain the surface pH as adjacent to neutral as feasible because an acidic or basic pH may bother the buccal mucosa. The gel-based sample was kept in contact with 5 mL of phosphate buffer containing 2% *w*/*v* agar medium (pH 6.8 ± 0.01) for 2 h at room temperature in order to cause it to swell. The electrode was placed in contact with the surface of the formulations, and the pH was then determined after one minute of equilibration [29,31]. The average of three readings was noted.

### 4.4. Swelling Index (SI)

The gravimetric method was used to determine the gel swelling index [32]. A 1% agar gel plate was used to measure the gel’s rate of swelling. The gel’s typical weight (W1) was computed. The formulations were put in a petri dish with a gel surface and kept at 37 ± 1 °C in an incubator. At one-hour intervals of time up to six hours, formulations were taken out, cleaned with regular filter paper, and reweighed (W2). The following formula was used to determine the index of swelling:

Index of swelling (SI) = [(W2 − W1)/W1] × 100

### 4.5. Ex Vivo Buccoadhesive Strength

The ex vivo buccoadhesive strength was assessed using a modified physical balance method [33,34]. Within two hours of the slaughter, fresh sheep buccal mucosa was obtained from a nearby slaughterhouse and employed. The underlying fat and loose tissues were cut out in order to separate the mucosal membrane. After being rinsed with distilled water, the membrane was then treated with phosphate buffer, pH 6.8. By maintaining a 5-g saliva solution at 37 °C prior to the investigation, the two sides of the balance were made equal. After being divided into pieces, the sheep buccal mucosa was cleaned with phosphate buffer, pH 6.8. The glass vial containing the phosphate buffer was connected to a piece of buccal mucosa. A glass vial was fitted tightly with a glass beaker that held phosphate buffer at pH 6.8 at 37 °C that barely touched the mucosal surface. The buccal gel weighs down the right-hand pan because it was attached to the bottom of a rubber stopper with cyanoacrylate adhesive. The right pan has 5 g of weight removed from it. This brought the pan and gel down over the mucosa. The balance was held in this posture for the entire five-minute contact period. Using an infusion device, water was gradually infused at a rate of 100 drops per minute (weight-equivalent) until the gel came away from the mucosal layer to the right-hand pan. The mucoadhesive strength of the buccal gel is expressed in grams by this detachment force:

Force of adhesion (N) = (Bioadhesive strength (g) × 9.8)/1000

Bond strength (N m^−2^) = Force of adhesion/surface area

### 4.6. In Vitro Drug Release Study

The drug release from the bilayer gel was investigated using the USP Type II rotating paddle method [35,36]. The dissolution medium was 900 mL of pH 6.8 phosphate buffer. At a rotational speed of 50 rpm and a temperature of 37 ± 1 °C, the release research was conducted. Cyanoacrylate adhesive was used to secure the buccal gel’s backing layer to the glass slide. The disc was put in the dissolution vessel’s bottom. To maintain sink conditions, aliquots (5 mL each) were taken out at regular intervals and refilled with fresh medium. Filtered samples were then subjected to appropriate phosphate buffer pH 6.8 dilutions and spectrophotometric analysis at 236 nm.

### 4.7. Ex Vivo Permeation Studies

Fresh sheep buccal mucosa was used in an ex vivo diffusion investigation of Nifedipine formulations in a modified diffusion cell [37]. It was mounted between the donor and the receptor compartments with the fresh sheep buccal mucosa. The donor compartment was an open-ended cylinder to which sheep buccal mucosa was connected. The gel needed to be positioned so that it adhered to the mucosal membrane. Isotonic phosphate buffer with a pH of 6.8 was put inside the receptor compartment. The assembly was kept at 37 °C while being magnetically agitated. At regular intervals, samples were taken out in triplicate, and they were subjected to UV spectrophotometer analysis at 236 nm.

### 4.8. Ex Vivo Muco Irritation by Histological Examination

Using fresh sheep buccal mucosa obtained from a nearby abattoir shortly after slaughter (sheep buccal mucosa was used for the histological evaluation within 2 h), ex vivo muco irritations of Nifedipine buccal formulations (NT5) were carried out [38]. During ex vivo permeation testing, the best formulation was applied over the sheep buccal mucosa and put into contact with the polymeric gel-based matrix layer followed by an impermeable ethyl cellulose backing layer, and the used mucosa was then histologically examined. In order to assess the pathological alterations in tissue structure and cell morphology that were brought on by the administration of buccoadhesive formulations, histological analysis was conducted. The mucosa’s epithelial tissues were dehydrated with graded ethanol (60 to 100%), were rinsed with distilled water for up to an hour, and were then fixed in 10% neutral buffered formalin for two hours. After that, the mucosa received the usual xylene permeation treatment and liquid paraffin embedding. Paraffin-embedded samples were cut into 4 µm thick sections on a microtome using a disposable blade after 8 h of formalin fixation, and they were then quickly stained with eosin.

### 4.9. In Vivo Drug Release Study

We chose six male New Zealand white rabbits 10–12 weeks old and weighing 2.5–3 kg. A healthy rabbit weighing between 2.5 and 3 kg was selected, and its health was examined for any ailment [39,40]. The rabbit was not in a dorsal position, since the forelimbs and the hind limbs were locked into the iron rod of the miniature operating table. With the aid of the forceps, the prepared buccoadhesive bilayer gel was inserted into the buccal membrane (cheek pouch). Dextrose solutions were regularly administered during the research period. To stop blood clotting, 1 mL of blood was periodically drawn using a syringe that also contained 1 mL of heparin solution. For around 30 min, these blood samples were centrifuged at 2500 rpm. After appropriate dilution, one ml of the supernatant was collected for spectrophotometric analysis at 236 nm. The resultant number represents the quantity of the drug that was released from the rabbit buccal mucosa.

### 4.10. In Vitro—In Vivo Correlation

Nifedipine release was compared using in vitro and in vivo correlations. This release is influenced by variables connected to the drug’s in vivo and in vitro properties [41,42]. Plots were made depicting the cumulative percentage of drug release both in vitro and in vivo. Saliva samples were collected and filtered from ten people aged between 18 and 40. The formulations from the finest batch were placed in separate Petri dishes containing 5 mL of human saliva, and they were stored in a 37 ± 0.2 °C oven for six hours [43,44]. At regular intervals, the appearance of the buccoadhesive formulations, such as the colour and the shape, and the Nifedipine concentration were both evaluated in order to determine their stability.

### 4.11. Stability Study

The formulation NT5 was chosen, and stability tests were conducted in desiccators under accelerated conditions of 40 °C and 75° RH. The samples were stored in the aforementioned settings for three months after being placed in amber-coloured screw-cap containers [45]. The physical characteristics, buccoadhesive strength, and in vitro drug release were evaluated on a regular basis. A one-way ANOVA test was used to analyse the results, followed by Tukey’s test. At *p* 0.05, the differences were deemed to be statistically significant.

## Figures and Tables

**Table 1:** Physicochemical evaluation of buccoadhesive bilayer formulations of nifedipine.

Formulation Code	Thickness (mm) ± SD	Weight Variation mg ± SD	Hardness (Kg/cm^2^)	Friability (%)	Drug Content In mg	Surface pH ± SD
NT1	2.38 ± 0.03	149 ± 0.52	4.1 ± 0.12	0.78 ± 0.03	19.91 ± 0.41	6.78 ± 0.06
NT2	2.39 ± 0.02	149 ± 0.76	4.0 ± 0.21	0.69 ± 0.03	19.85 ± 0.19	6.76 ± 0.03
NT3	2.39 ± 0.03	150 ± 0.69	4.1 ± 0.32	0.67 ± 0.04	20.32 ± 0.21	6.68 ± 0.02
NT4	2.38 ± 0.05	149 ± 0.74	4.0 ± 0.28	0.65 ± 0.04	20.26 ± 0.41	6.76 ± 0.04
NT5	2.42 ± 0.03	150 ± 0.22	4.2 ± 0.24	0.59 ± 0.01	20.02 ± 0.15	6.80 ± 0.02
NT6	2.38 ± 0.04	149 ± 0.89	4.1 ± 0.24	0.84 ± 0.02	20.19 ± 0.01	6.75 ± 0.06
NT7	2.39 ± 0.07	149 ± 0.98	4.1 ± 0.36	0.67 ± 0.04	19.78 ± 0.22	6.69 ± 0.06
NT8	2.40 ± 0.02	149 ± 0.76	4.3 ± 0.29	0.63 ± 0.03	19.79 ± 0.65	6.72 ± 0.06
NT9	2.38 ± 0.02	150 ± 0.87	4.2 ± 0.34	0.72 ± 0.01	20.21 ± 0.31	6.75 ± 0.04
NT10	2.41 ± 0.02	150 ± 0.26	4.1 ± 0.51	0.74 ± 0.03	20.15 ± 0.15	6.79 ± 0.04

**Table 2:** Stability studies of NT5.

Parameters	1st Month	2nd Month	3rd Month	*p* Value
Physical appearance	No Change	No Change	No Change	-
Buccoadhesive strength	34.88 ± 1.09 ^ns^	35.3 ± 1.09 ^ns^	36 ± 0.34 ^ns^	0.1539
In vitro drug release	98.06 ± 0.55 ^ns^	98.13 ± 0.32 ^ns^	98.26 ± 0.5 ^ns^	0.8709

All values are expressed as Mean ± SD of triplicate measurements; ^ns^ = non-significant.

**Table 3:** The buccoadhesive bilayer formulations of Nifedipine.

Formulation Code		NT1	NT2	NT3	NT4	NT5	NT6	NT7	NT8	NT9	NT10
**Ingredients (mg)**	Nifedipine	20	20	20	20	20	20	20	20	20	20
	HPMC K 100	25	-	12.5	12.5	25	-	6.25	25	6.25	37.5
	SCMC	12.5	25	-	25	-	12.5	6.25	6.25	25	-
	PVP K 30	-	12.5	25	-	12.5	25	25	6.25	6.25	-
	CP 934	10	10	10	10	10	10	10	10	10	10
	Mg. stearate	2.5	2.5	2.5	2.5	2.5	2.5	2.5	2.5	2.5	2.5
	Lactose	15	15	15	15	15	15	15	15	15	15
	Mannitol	15	15	15	15	15	15	15	15	15	15
	EC	50	50	50	50	50	50	50	50	50	50
Total weight in mg		150	150	150	150	150	150	150	150	150	150

## Data Availability

The data presented in this study are available on request from the corresponding author.

## Supplementary Materials

The following supporting information can be downloaded at: https://www.mdpi.com/article/10.3390/gels9090688/s1, Figure S1: DSC curve of Nifedipine; Figure S2: DSC curve of HPMC K 100; Figure S3: DSC curve of SCMC; Figure S4: DSC curve of Carbopol 934; Figure S5: DSC curve of Nifedipine and polymer mixture; Figure S6: Controlled buccal mucosa; Figure S7: Test buccal mucosa; Table S1: Swelling index data for all formulations; Table S2: Ex-vivo buccoadhesive strength; Table S3: In-vitro and Higuchi’s release data; Table S4: Peppa’s data; Table S5: Ex-vivo permeation data of NT5; Table S6: In-vivo drug release of NT5; Table S7: In-vitro and In-vivo correlation data of NT5.
