# Supplementary material for: Development and Characterization of Gel-Based Buccoadhesive Bilayer Formulation of Nifedipine
Source: Gels. 2023 Aug 26;9(9):688. doi: 10.3390/gels9090688 (PMC10530715; doi:10.3390/gels9090688)
Supplement: Supplementary file 1 [file gels-09-00688-s001.zip › gels-2535170-supplementary.pdf]

# Supplementary Information

# Development and Characterization of Gel-Based Buccoadhesive Bilayer Formulation of Nifedipine

**M. Alagusundaram [1,*], Nem Kumar Jain [2], M. Yasmin Begum [3,*], S. Angala Parameswari [4], Vinod Kumar Nelson [5], Mohammad F. Bayan [6] and Balakumar Chandrasekaran [6]**

[1] Department of Pharmaceutics, School of Pharmacy, ITM University, Gwalior 474001, Madhya Pradesh, India
[2] Department of Pharmacology, School of Pharmacy, ITM University, Gwalior 474001, Madhya Pradesh, India; nemjain.pharma@itmuniversity.ac.in
[3] Department of Pharmaceutics, College of Pharmacy, King Khalid University, Abha 61421, Saudi Arabia
[4] Department of Pharmaceutical Analysis, Jagan's Institute of Pharmaceutical Sciences, Nellore 524346, Andhra Pradesh, India; eswarialagusundaram@gmail.com
[5] Department of Pharmaceutical Chemistry, Raghavendra Institute of Pharmaceutical Education and Research, Anantapuramu 515721, Andhra Pradesh, India; vinod.kumar457@gmail.com
[6] Faculty of Pharmacy, Philadelphia University, P.O. Box 1, Amman 19392, Jordan; mbayan@philadelphia.edu.jo (M.F.B.); balakumar@philadelphia.edu.jo (B.C.)
* Correspondence: alagusundaram.sop@itmuniversity.ac.in (M.A.); ybajen@kku.edu.sa (M.Y.B.)

## Table of Contents


| S. No. | Contents | Page Number |
|---|---|---|
| 1 | Figure S1. DSC curve of Nifedipine | 3 |
| 2 | Figure S2. DSC curve of HPMC K 100 | 3 |
| 3 | Figure S3. DSC curve of SCMC | 4 |
| 4 | Figure S4. DSC curve of Carbopol 934 | 4 |
| 5 | Figure S5. DSC curve of Nifedipine and polymer mixture | 5 |
| 6 | Figure S6. Controlled buccal mucosa | 5 |
| 7 | Figure S7. Test buccal mucosa | 6 |
| 8 | Table S1. Swelling index data for all formulations | 6 |
| 9 | Table S2. Ex-vivo buccoadhesive strength | 7 |
| 10 | Table S3. In-vitro and Higuchi's release data | 7 |
| 11 | Table S4. Peppa's data | 8 |
| 12 | Table S5. Ex-vivo permeation data of NT5 | 9 |
| 13 | Table S6. In-vivo drug release of NT5 | 9 |
| 14 | Table S7. In-vitro and In-vivo correlation data of NT5 | 10 |

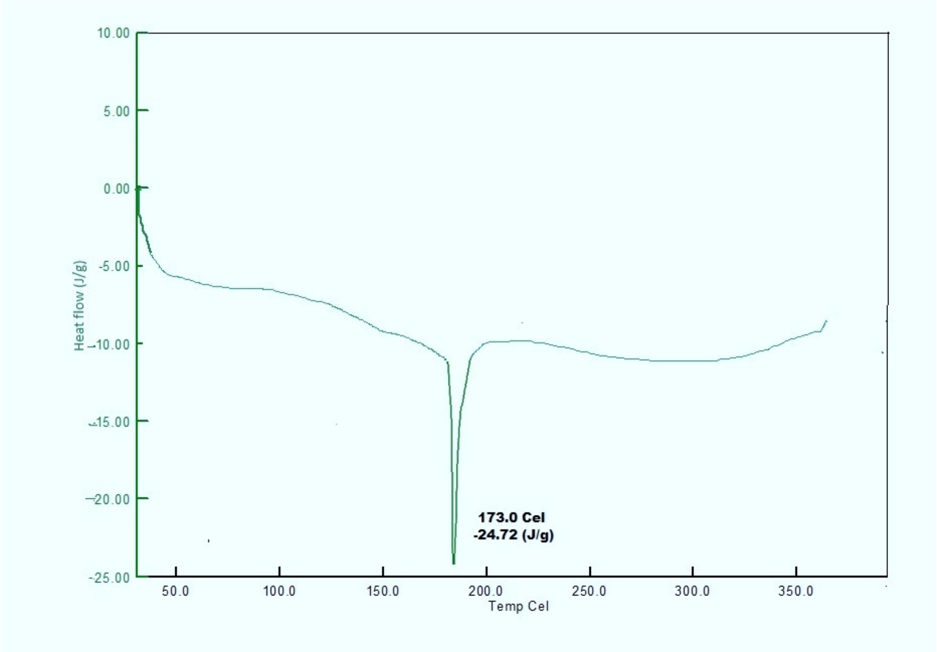


**Figure S1**. DSC curve of Nifedipine

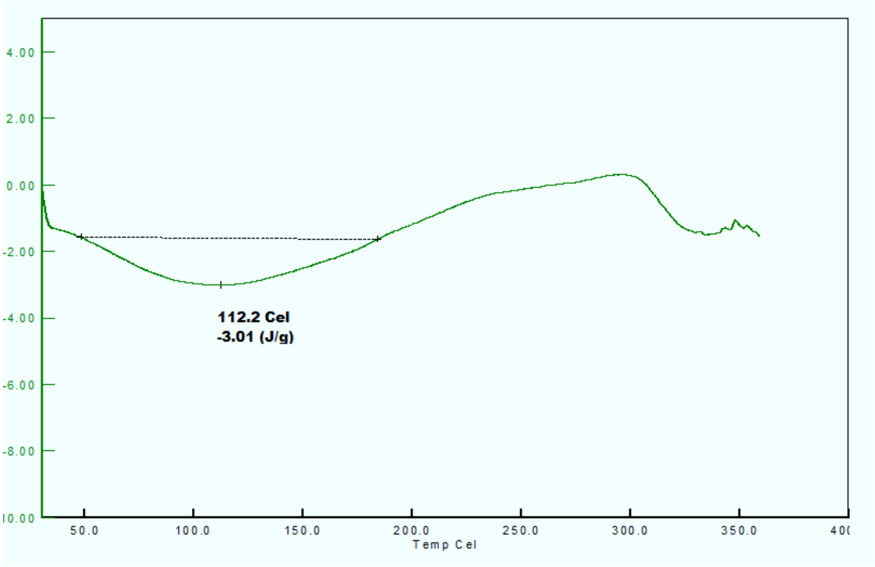


**Figure S2**. DSC curve of HPMC K 100

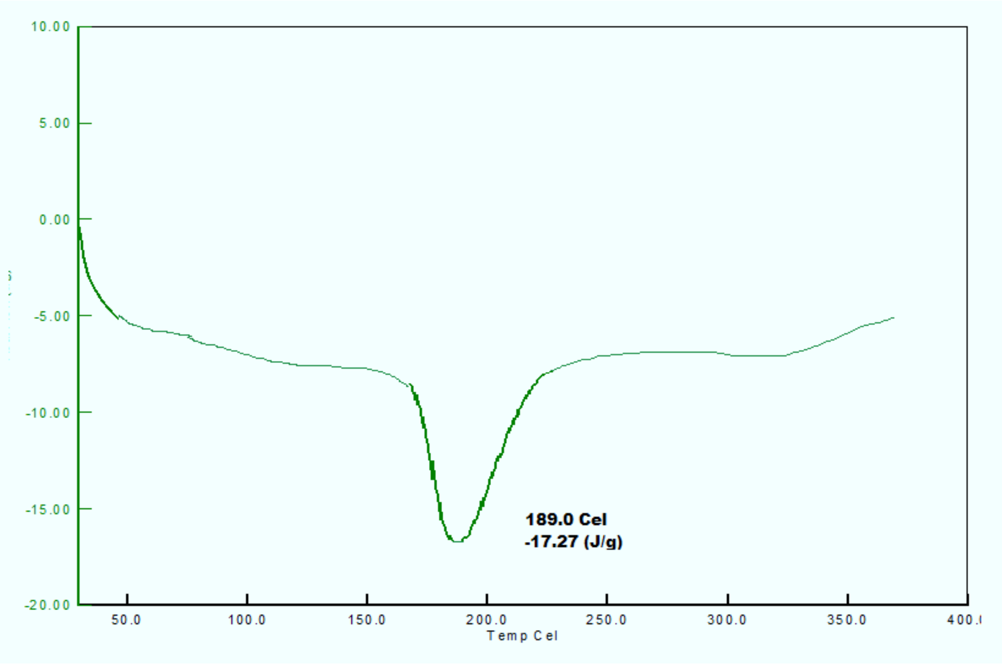


**Figure S3**. DSC curve of SCMC

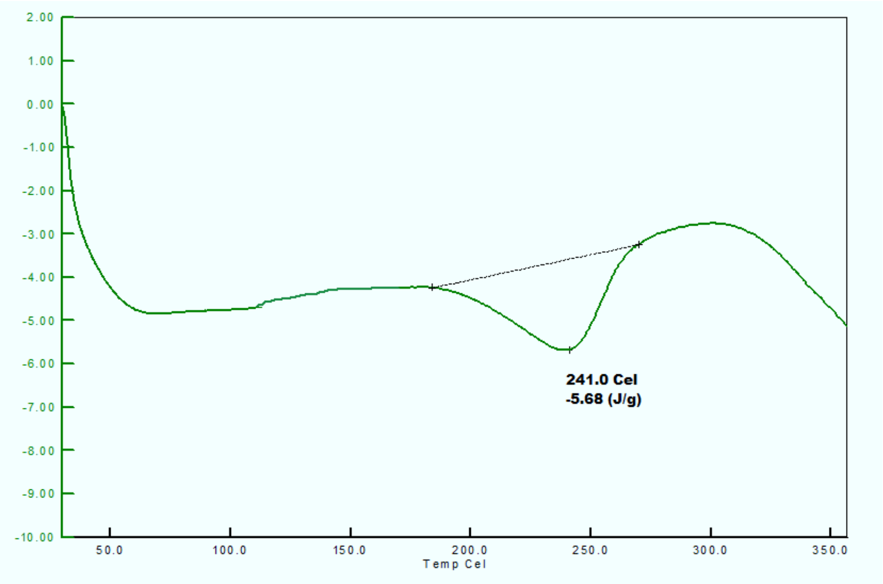


**Figure S4**. DSC curve of Carbopol 934

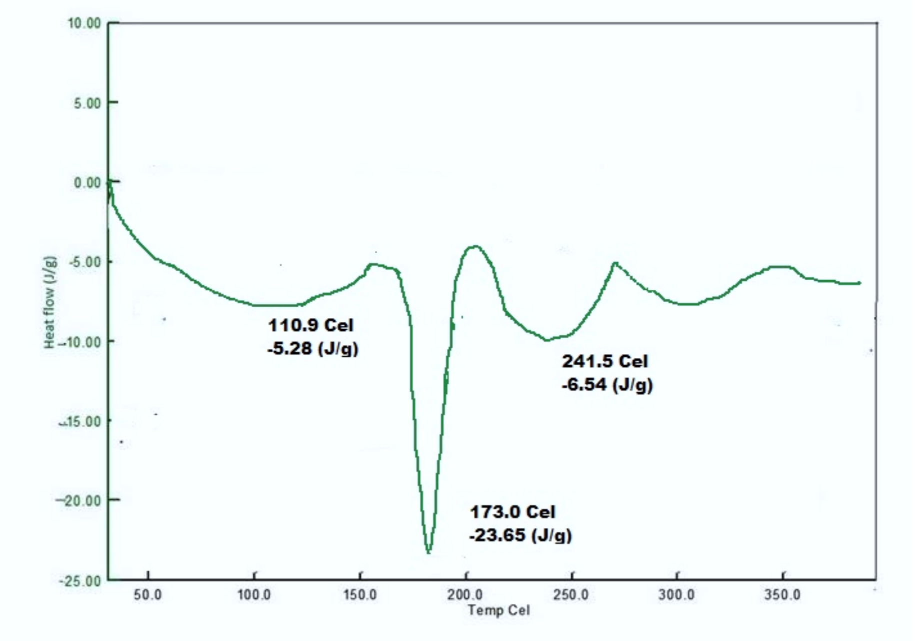


**Figure S5**. DSC curve of Nifedipine and polymer mixture

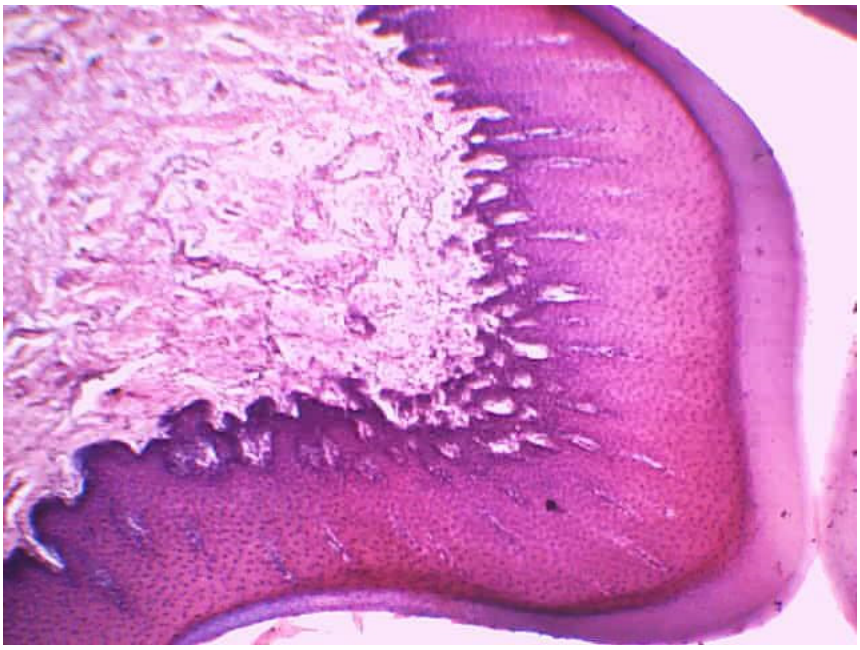

**Figure S6**. Controlled buccal mucosa

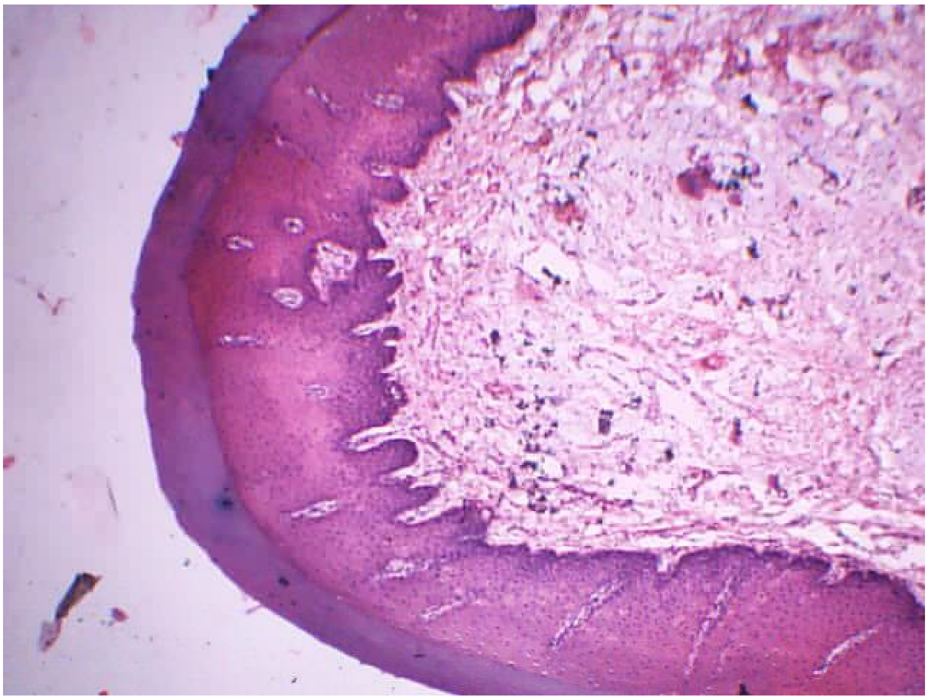

**Figure S7**. Test buccal mucosa

**Table S1. Swelling index data for all formulations**

| Formulation code | Swelling index ±S.D | | | | | |
|---|---|---|---|---|---|---|
| | Time in h | | | | | |
| | 1 | 2 | 3 | 4 | 5 | 6 |
| NT1 | 26.09±0.76 | 38.61±1.08 | 55.58±0.80 | 64.96±0.70 | 71.27±0.76 | 74.84±0.27 |
| NT2 | 22.23±0.72 | 32.15±0.91 | 40.75±0.46 | 50.71±0.54 | 60.04±0.61 | 65.21±0.53 |
| NT3 | 19.19±0.64 | 24.48±0.63 | 37.81±0.67 | 45.84±0.68 | 51.8±0.66 | 55.77±0.51 |
| NT4 | 23.73±1.08 | 33.97±0.48 | 46.13±0.93 | 51.81±0.69 | 63.84±0.28 | 68.91±0.93 |
| NT5 | 27.39±1.03 | 41.62±0.90 | 57.67±0.53 | 66.68±0.75 | 71.25±0.61 | 78.6±1.04 |
| NT6 | 19.81±0.67 | 31.39±0.98 | 39.81±0.67 | 51.12±0.62 | 57.52±1.08 | 62.76±0.43 |
| NT7 | 16.01±0.84 | 25.64±0.75 | 32.76±0.54 | 41.10±0.88 | 46.46±0.87 | 51.76±0.64 |
| NT8 | 26.65±0.72 | 40.98±0.79 | 56.93±0.86 | 65.29±0.97 | 71.28±0.30 | 74.84±0.60 |
| NT9 | 23.35±1.12 | 31.43±0.64 | 41.91±0.93 | 51.66±0.57 | 61.44±0.63 | 65.69±0.64 |
| NT10 | 31.47±0.93 | 42.62±0.77 | 58.41±0.79 | 67.45±0.96 | 73.17±0.61 | 76.85±0.65 |

**Table S2. Ex-vivo buccoadhesive strength**

| Formulation code | Buccoadhesive Strength in g |
|---|---|
| NT1 | 33.2 |
| NT2 | 22.6 |
| NT3 | 23.5 |
| NT4 | 26.4 |
| NT5 | 34.6 |
| NT6 | 19.2 |
| NT7 | 22.1 |
| NT8 | 33.8 |
| NT9 | 23.4 |
| NT10 | 32.7 |

**Table S3. In-vitro and Higuchi's release data**

| TIME | Sqrt | NT1 | NT2 | NT3 | NT4 | NT5 | NT6 | NT7 | NT8 | NT9 | NT10 |
|---|---|---|---|---|---|---|---|---|---|---|---|
| 0 | 0 | 0 | 0 | 0 | 0 | 0 | 0 | 0 | 0 | 0 | 0 |
| 1 | 1 | 10.8 | 12.6 | 11.8 | 13.4 | 12.8 | 11.2 | 14.2 | 10.2 | 13.2 | 11.6 |
| 2 | 1.41421 | 18.2 | 23.2 | 21.6 | 22.8 | 19.2 | 20.6 | 25.6 | 18.6 | 23.8 | 18.4 |
| 3 | 1.73205 | 26.1 | 31.4 | 32.8 | 33.2 | 28.8 | 31.2 | 37.1 | 25.2 | 32.4 | 24.6 |
| 4 | 2 | 34.2 | 42.6 | 43.2 | 45.1 | 36.2 | 42.4 | 46.5 | 32.8 | 43.2 | 32.2 |
| 5 | 2.23607 | 43.2 | 49.8 | 52.1 | 53.6 | 43.4 | 48.6 | 54.8 | 42.6 | 51.2 | 43.8 |
| 6 | 2.44949 | 51.4 | 57.6 | 63.6 | 64.2 | 50.6 | 56.8 | 66.6 | 52.4 | 59.4 | 51.6 |
| 7 | 2.64575 | 62.2 | 68.2 | 70.6 | 71.9 | 61.2 | 67.4 | 75.2 | 60.8 | 68.3 | 61.8 |
| 8 | 2.82843 | 71.6 | 74.6 | 78.4 | 79.2 | 69.6 | 73.2 | 83.4 | 71.6 | 76.2 | 72.2 |
| 9 | 3 | 81.2 | 81.2 | 83.9 | 84.4 | 78.2 | 80.6 | 90.2 | 82.3 | 81.8 | 79.4 |
| 10 | 3.16228 | 87.4 | 89.6 | 91.2 | 92.4 | 86.4 | 88.2 | 97.4 | 87.2 | 89.4 | 86.2 |
| 11 | 3.316625 | 92.4 | 97.1 | 97.4 | 98.6 | 92.8 | 97.6 | | 91.6 | 98.2 | 91.3 |
| 12 | 3.464102 | 98.3 | | | | 98.6 | | | 98.4 | | 98.1 |

## Table S4. Peppa's data

| **LOG T** | **LOG % R** | | | | | | | | | |
|---|---|---|---|---|---|---|---|---|---|---|
| | NT1 | NT2 | NT3 | NT4 | NT5 | NT6 | NT7 | NT8 | NT9 | NT10 |
| 0 | 1.03342 | 1.10037 | 1.07188 | 1.1271 | 1.10721 | 1.04922 | 1.15229 | 1.0086 | 1.12057 | 1.06446 |
| 0.30103 | 1.26007 | 1.36548 | 1.33445 | 1.35793 | 1.2833 | 1.31386 | 1.40824 | 1.26951 | 1.37657 | 1.26482 |
| 0.477121 | 1.41664 | 1.49693 | 1.51587 | 1.52114 | 1.45939 | 1.49415 | 1.56937 | 1.4014 | 1.51054 | 1.39093 |
| 0.60206 | 1.53403 | 1.62941 | 1.63548 | 1.65417 | 1.55871 | 1.62736 | 1.66745 | 1.51587 | 1.63548 | 1.50785 |
| 0.69897 | 1.63548 | 1.69723 | 1.71684 | 1.72916 | 1.63749 | 1.68664 | 1.73878 | 1.62941 | 1.70926 | 1.64147 |
| 0.778151 | 1.71096 | 1.76042 | 1.80346 | 1.80753 | 1.70415 | 1.75434 | 1.82347 | 1.71933 | 1.77378 | 1.71265 |
| 0.845098 | 1.79379 | 1.83378 | 1.8488 | 1.85673 | 1.78675 | 1.82865 | 1.87622 | 1.7839 | 1.83442 | 1.79099 |
| 0.90309 | 1.85491 | 1.87274 | 1.89432 | 1.89872 | 1.84261 | 1.86451 | 1.92117 | 1.85491 | 1.88195 | 1.85854 |
| 0.954243 | 1.90956 | 1.90955 | 1.92376 | 1.92634 | 1.89321 | 1.90633 | 1.95521 | 1.91539 | 1.91275 | 1.89982 |
| 1 | 1.94151 | 1.95231 | 1.95999 | 1.96567 | 1.93651 | 1.94547 | 1.98856 | 1.94052 | 1.95134 | 1.93551 |
| 1.041393 | 1.96567 | 1.98722 | 1.98856 | 1.99387 | 1.96755 | 1.98945 | | 1.96189 | 1.99211 | 1.96047 |
| 1.079181 | 1.99255 | | | | 1.99388 | | | 1.99299 | | 1.99167 |

**Table S5. Ex-vivo permeation data of NT5**

| Time (h) | Cumulative % Drug Release |
|---|---|
| 0 | 0 |
| 1 | 7.24 |
| 2 | 13.15 |
| 3 | 22.18 |
| 4 | 29.41 |
| 5 | 41.26 |
| 6 | 50.42 |
| 7 | 57.6 |
| 8 | 63.75 |
| 9 | 71.32 |
| 10 | 78.24 |
| 11 | 86.53 |
| 12 | 93.81 |

**Table S6. In-vivo drug release of NT5**

| Time in h | Cumulative percentage drug release |
|---|---|
| **0** | **0** |
| 1 | 9.62 |
| 3 | 23.48 |
| 6 | 44.69 |
| 9 | 67.84 |
| 12 | 90.74 |

**Table S7. In-vitro and In-vivo correlation data of NT5**

| Time in h | *In-vivo* cumulative % drug release | *In-vitro* cumulative % drug release |
|---|---|---|
| | ***0*** | ***0*** |
| 1 | 9.62 | 12.8 |
| 3 | 23.48 | 28.8 |
| 6 | 44.69 | 50.6 |
| 9 | 67.84 | 78.2 |
| 12 | 90.74 | 98.6 |
